# Usefulness of pT1 substaging in papillary urothelial bladder carcinoma

**DOI:** 10.1186/s13000-016-0466-6

**Published:** 2016-01-20

**Authors:** Carlo Patriarca, Rodolfo Hurle, Marco Moschini, Massimo Freschi, Piergiuseppe Colombo, Maurizio Colecchia, Lucia Ferrari, Giorgio Guazzoni, Andrea Conti, Giario Conti, Roberta Lucianò, Tiziana Magnani, Renzo Colombo

**Affiliations:** Department of Pathology, Azienda Ospedaliera Sant’Anna, 22020 Como, Italy; Department of Urology, Humanitas Research Hospital, Rozzano (MI), Italy; Department of Urology, Ospedale San Raffaele, Milan, Italy; Department of Pathology, Ospedale San Raffaele, Milan, Italy; Department of Pathology, Humanitas Research Hospital, Rozzano (MI), Italy; Department of Pathology, Fondazione IRCCS Istituto Nazionale dei Tumori, Milan, Italy; Department of Urology, Fondazione IRCCS Istituto Nazionale dei Tumori, Milan, Italy; Department of Urology, Azienda Ospedaliera Sant’Anna, Como, Italy

**Keywords:** Urothelial bladder carcinoma, Stage, Substaging system, Prognosis, Progression

## Abstract

**Background:**

When treating bladder cancer patients, the most significant problems usually concern cases with high-grade non-muscle-invasive carcinoma, and a better understanding of which patients would benefit from early radical cystectomy is urgently needed. The uropathology community is seeking more user-friendly approaches to distinguishing between T1 cancers exhibiting different types of clinical behavior.

**Methods:**

After a retrospective review, we selected a group of 314 patients who underwent transurethral resection of the bladder (TURB) and were diagnosed with high-grade urothelial carcinoma staged as T1. Three different substaging systems were applied: one was the anatomy-based T1 a/b; and two involved micrometric thresholds of either 0.5 mm of invasion (as proposed by van Rhijn et al.), or 1 mm of invasion (as proposed in the present study). Early reTUR (repeated transurethral resection) was performed in 250 patients, and the same substaging approaches were applied to cases of T1.

**Results:**

It proved feasible to apply the 1 mm substaging system in 100 % of cases, the van Rhijn system in 100 %, and the anatomy-based method (T1 a/b) in 72.3 % of cases. At a mean follow-up of 46 months, the recurrence-free survival rate was significantly better (*p* < 0.001) in the group that underwent reTUR, while none of the three substaging systems reliably predicted recurrences. The 1 mm did seem promising, however, as a threshold for predicting progression, reaching statistical significance in the Kaplan Meier estimates (*p* < 0.04).

**Conclusion:**

Our study shows that micrometric substaging is feasible in this setting and should be extended to include any early reTUR to complete the substaging done after the first TURB. It can also provide helpful prognostic information.

## Background

Urothelial carcinoma poses a major challenge for Western health care systems, being the seventh most frequent cancer and one of the most expensive malignancies to diagnose and manage [1], making any improvement to the strategy for diagnosing and treating this disease important to the medical and scientific community. Non-muscle-invasive bladder carcinoma (NMIBC) accounts for about 75 % of bladder cancers, and high-grade T1 is the subtype of NMIBC at highest risk, with patients facing long-term cancer-specific mortality rates as high as 34 % [2]. Due to the impact of this category of patients, in terms of disease incidence and patient survival, there are many important clinical and surgical factors to consider when deciding the best treatment for T1 bladder carcinoma, that also concern the precise role of early cystectomy. Issues such as age, gender, tumor size, number of lesions and time to relapse all have an important role in the primary treatment of T1 tumors and after first-line BCG treatment has failed [3]. Pathological factors such as grade [1], histotype, or histological variant [4], tumor growth pattern [5], lymphovascular invasion [6], and association with in situ carcinoma [7] also play a central part in orienting towards the most appropriate clinical management of patients.

Despite the lively debate and active research on this topic, whether or not to opt for radical cystectomy is sometimes still a dilemma, since it is unclear which patients might suffer an adverse outcome. Further prognostic morphophenotypic information is much needed. Although the substaging of the T1 tumors is neither recommended by the WHO/ISUP, nor included in the VII edition of the TNM [8], one of the pathological details most often requested by clinicians is “some form of estimate of lamina propria invasion in pT1 tumors”, as recently mentioned in Mahul Amin et al. [9]. Many different systems have consequently been proposed in the literature in the past few years, but none have been wholly satisfactory. Defining cutoffs for pT1 substaging has proved challenging. The most widely applied are histopathological parameters based on invasion of the *muscularis mucosae* - i.e. within the lamina propria above the *muscularis mucosae* (T1a), within the *muscularis mucosae* (T1b), and beyond the *muscularis mucosae* (T1c) [10]; or, more simply, above or into *vs* beyond the *muscularis mucosae* (T1 a/b) [11]. The main weakness of this anatomy-based system lies in that it suffers from a limited feasibility of application and a poor reproducibility. Another way to distinguish between pT1 tumors is to measure the maximum extent of invasion at any direction [12]. Using a size-based approach, van Rhijn et al. [13] established a clinically significant threshold of invasion at 1 high-power field (HPF 40x) and divided their cohort of T1 invasive bladder tumors into pT1m (microinvasive) and pT1e (extensively invasive). The same approach and substaging threshold were adopted by Bertz et al. [14]. The aim of the present study is to test in TUR plus early reTUR specimens a system (called ROL, from *Rete Oncologica Lombarda*) based on a different threshold of microscopically identifiable invasion in a larger group of patients than in the study conducted by van Rhijn et al., assessing the feasibility and clinical relevance of this substaging approach as an oncological outcome predictor, and to compare the ROL with the anatomy-based T1a/b and the HPF-based T1m/e substaging methods.

## Methods

Five pathologists (CP, LF, MF, PGC, MC) from four different high-volume urology referral centers retrospectively examined multiple slides of a total of 450 transurethral resections of the bladder (TURB) from patients with an original diagnosis of T1 high-grade papillary urothelial carcinoma (tumors graded according to the WHO 2004 Classification). The study was performed in accordance with the code of conduct of the medical scientific societies and based on approval of institutional review boards. This sample was selected from among 502 such cases, 52 of which were not included in the study because they were staged as T2 on early reTUR (i.e. the second resection of the tumor bed performed soon after TURB to ensure that *muscularis propria* was obtained). The cases were gathered from consecutive patients undergoing TURB, retracing the files from 2011 to 2007, at the Urology Departments of the St Anna Hospital (Como), San Raffaele Hospital (Milano), Humanitas Research Hospital (Rozzano, Milano) and National Cancer Institute (Milano), Italy.

Of the 450 cases considered, 314 (69 %) were confirmed as T1 high-grade urothelial carcinomas, while 136 (31 %) were excluded from the study due to erroneous grading, revised staging (cases downstaged to Ta or upstaged to T2), or, more often, because patients were lost to follow-up. Other histotypes (small cell or adenocarcinoma, or squamous cell carcinoma) were also ruled out. The microscopic criteria used to define invasion were as established in the literature [15], and the substaging rules adopted were discussed and approved by all co-authors. In particular, a cutoff of 1 mm of invasion was adopted (we called this substaging approach ROL [*Rete Oncologica Lombarda*], after Lombardy Oncology Network) because the 0.5 mm threshold clinically tested in another study (van Rhijn et al.; 13) was too superficial in the opinion of the present authors, partly because of the uneven thickness of the lamina propria, which can reach as much as 3 mm in the cupola [16]. Our ROL substaging was thus defined as follows: ROL1 < 1 PF (objective 20x, ocular 10x/field 22, diameter 1.1 mm) of invasion, approximately corresponding to invasion of the lamina propria 1 mm thick or less; ROL2: > 1 PF (objective 20x), approximately corresponding to invasion of the lamina propria more than 1 mm thick, *or* multifocal invasion with foci cumulatively amounting to invasion of the lamina propria more than 1 mm thick. We measured the major diameter of invasion, or extent of invasion in any direction. Cases were also anatomically defined as T1a/T1b (above or into *vs* below the *muscularis mucosae*) [11]. In the absence of the *muscularis mucosae*, the edge of the invasive tumor was compared with the vascular plexus of the lamina propria. The van Rhijn substaging method was applied too [13], i.e. < 1 HPF (objective 40x, ocular 10x/field 22, diameter of 0.55 mm) of lamina propria invasion, approximately corresponding to 0.5 mm of invasion of the lamina propria or less (called T1m) versus > 1 HPF (objective 40x), approximately corresponding to more than 0.5 mm of invasion of the lamina propria, *or* multifocal invasion without combining the thickness of individual foci (called T1e). Any presence of carcinoma in situ (CIS) or neoplastic vascular invasion was also recorded.

To test the reproducibility of the substaging systems, a set of the first 50 consecutive cases was blindly reviewed by all the pathologists and, in the event of differences of opinion - 2/50 (4 %) for the ROL, and 1/50 (2 %) for the T1m/e substaging - cases were discussed to reach a consensus.

Of the 314 patients considered, 250 (79 %) underwent repeat TURB (reTUR) within three months of their first TURB: 141 (56 %) were T0 patients, 40 (16 %) were Ta, 57 (22 %) were T1, 10 (4 %) were CIS, and 2 (0.8 %) were case of flat dysplasia. Patients diagnosed as T1 at reTUR were also reviewed by all the pathologists using the same substaging thresholds, and the residual foci of infiltration were added to those of the original TUR to obtain a complete evaluation for substaging purposes.

Clinical follow-up data were collected on all patients, including disease recurrences and progression. The median follow-up was 46 months. All patients except those who underwent cystectomy immediately were treated with BCG.

Recurrence was defined as a tumor recurring with a lower or the same stage as the primary tumor, and progression as a tumor recurring with a higher stage or metastatic disease. The three substaging methods were compared in terms of feasibility and prognostic reliability. Progression-free survival (PFS) and recurrence-free survival (RFS) were analyzed by means of Kaplan Meier estimates. Univariate Cox’s regression analyses were used to test the risk factors associated with a detrimental effect on survival.

### Statistical analyses

Descriptive statistics of categorical variables focused on frequencies and proportions. Means, medians and interquartile ranges (IQR) are given for continuously coded variables. Univariate Cox’s proportional hazard models were constructed to identify significant predictors of recurrence and progression after TURB. Kaplan Meier analyses were plotted to compare different substaging methods, analyzing recurrence and progression. All analyses were performed using SPSS v. 20.0 (IBM Corp., Armonk, NY, USA).

## Results

Our study concerned 314 cases treated with TURB and confirmed as T1 high-grade urothelial carcinoma according to the WHO 2004 Classification, with a mean follow-up of 46 months. Patients were a mean 71.3 years of age (range 64–79), and 39 patients (12.4 %) were female (see Table 1 for the descriptive characteristics). The reTUR performed in 250 cases was positive in 107: 40 (16 %) were Ta, 57 (22 %) were T1, and 10 (4 %) were CIS. As mentioned earlier, we considered reTUR within 3 month as a completion of the original procedure for tumor staging purposes. Hence, residual T1 areas of infiltration of 57 cases were submitted to the same analysis reaching a final substaging. All patients were treated with BCG, except for those who underwent cystectomy immediately (52 patients, 16 %).

**Table 1:** Descriptive characteristics of 314 patients treated with transurethral resection for T1HG Bladder carcinoma between 2011 and 2007

Variables	Overall population (*n* = 314, 100 %)
Number recurrence	115 (36.6 %)
Number progression	33 (10.5 %)
Gender	
Male	275 (87.6 %)
Female	39 (12.4 %)
Age	
Mean	71.3
Median (IQR)	72 (64–79)
Recurrence (EAU classification)	
First episode	218 (69.4 %)
Recurrent episode	90 (28.7 %)
More than 1 episode	6 (1.9 %)
Focality	
1	140 (44.6 %)
2	24 (7.6 %)
3	66 (21.0 %)
No data	84 (26.8 %)
Pure transitional	281 (89.5 %)
Areas of divergent differentiation	33 (10.5 %)
CIS	54 (17.2 %)
LVI	33 (10.5 %)
SUB-STAGING	
MM level feasibility	230 (73.2 %)
van Rhijn system feasibility	314 (100 %)
ROL system feasibility	314 (100 %)
*Muscolaris Mucosae*	
Above (T1a)	177 (56.4 %)
Below (T1b)	51 (16.2 %)
van Rhijn	
T1m	109 (34.7 %)
T1e	205 (65.3 %)
ROL	
1	152 (48.4 %)
2	162 (51.6 %)
ROL FOCI	
2/3	31 (9.9 %)
> 3	99 (31.5 %)

*IQR* interquartile range, *CIS* carcinoma in situ, *LVI* lymphovascular invasion, *MM* muscolaris mucosa, *ROL* rete oncologica lombardia

In the original cohort of 314 patients, there were 90 cases of multifocal disease. CIS was identified in 54 cases (17.1 %), and vascular invasion in 33 (10.5 %). A papillary urothelial carcinoma histology was seen in 89.5 % of cases, and the focal variant in the other 11.5 % (33 cases showing focal adenocarcinoma or squamous cell carcinoma differentiation) (Table 1).

**Fig. 1:**
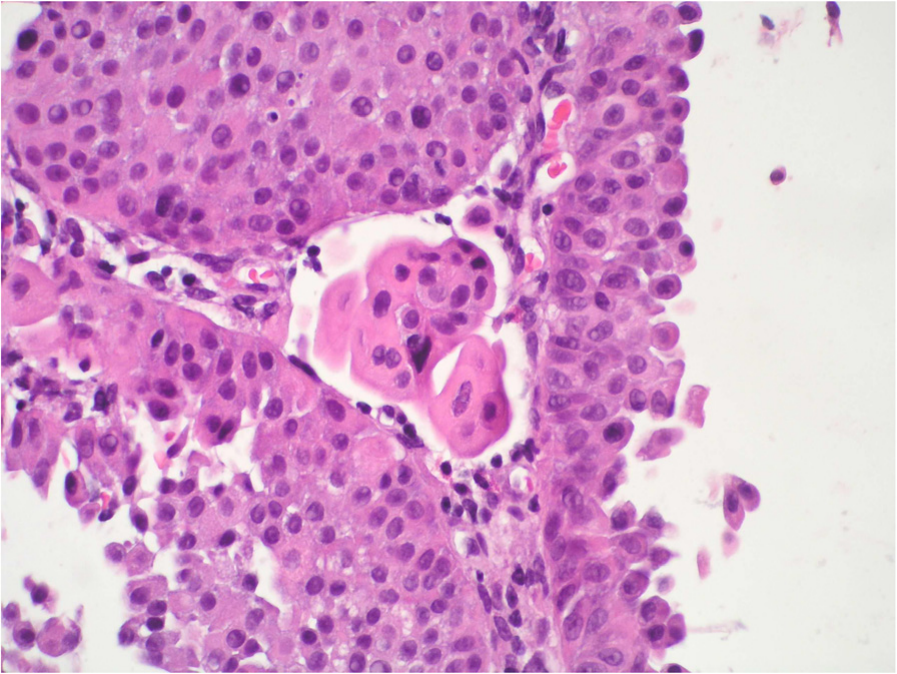
Lamina propria invasion in a high-grade papillary urothelial carcinoma: T1m/ROL1 substaging (T1 < 0.5 mm)

**Fig. 2:**
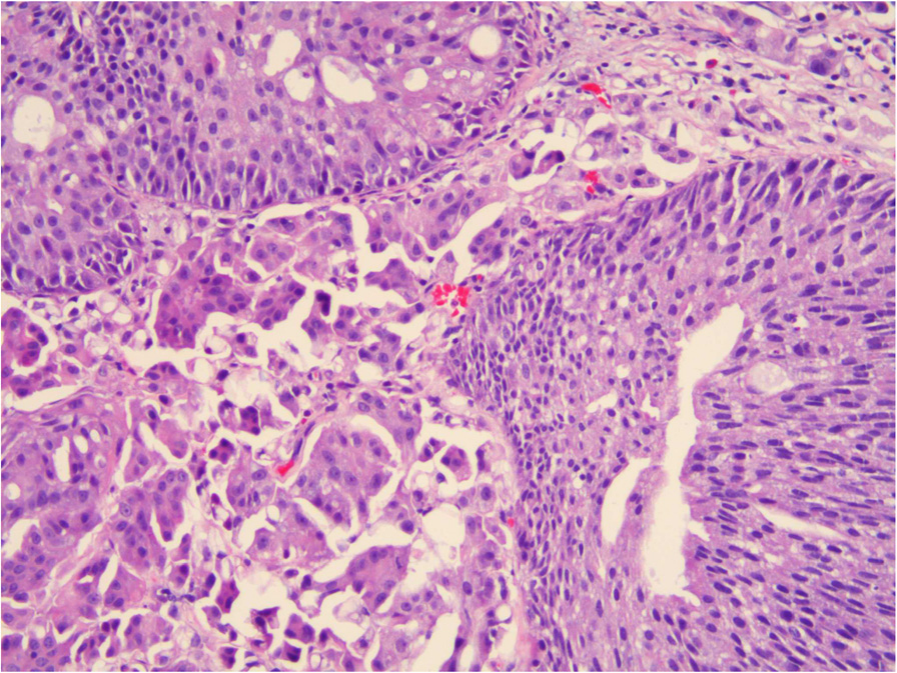
Lamina propria invasion in a high-grade papillary urothelial carcinoma: T1e/ROL1 substaging (the focus is contained within one x 200 field, although it is larger than one x400 field)

**Fig. 3:**
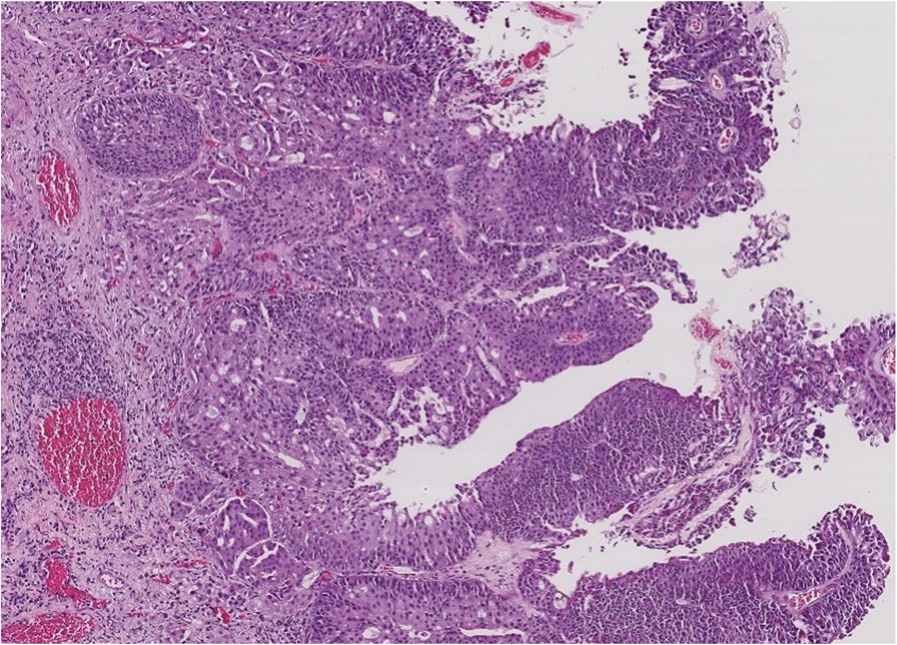
Multiple foci of lamina propria invasion in a high-grade papillary urothelial carcinoma: T1e/ROL2 substaging (multifocality of invasion cumulatively amounting to more than 1 mm, i.e. > 200x field)

As for the feasibility of substaging, the ROL system was feasible in 100 % of cases, the anatomy-based method (T1 a/b) in 72.3 %, and the van Rhijn system in 100 %. Figures 1, 2 and 3 show examples of histological applications of the ROL system compared with van Rhijn and T1a/b substaging. All the infiltrative patterns described in the literature were present, i.e. those characterized by a paradoxical differentiation of individual clusters or single cells, irregular infiltrative tongues and irregular small or large nests of infiltrative tumor with fibroinflammatory reactions. Cases revealing ambiguous or deceptive patterns of infiltration were excluded from the study and classified as Ta, as recommended in the literature [17].

**Fig. 4:**
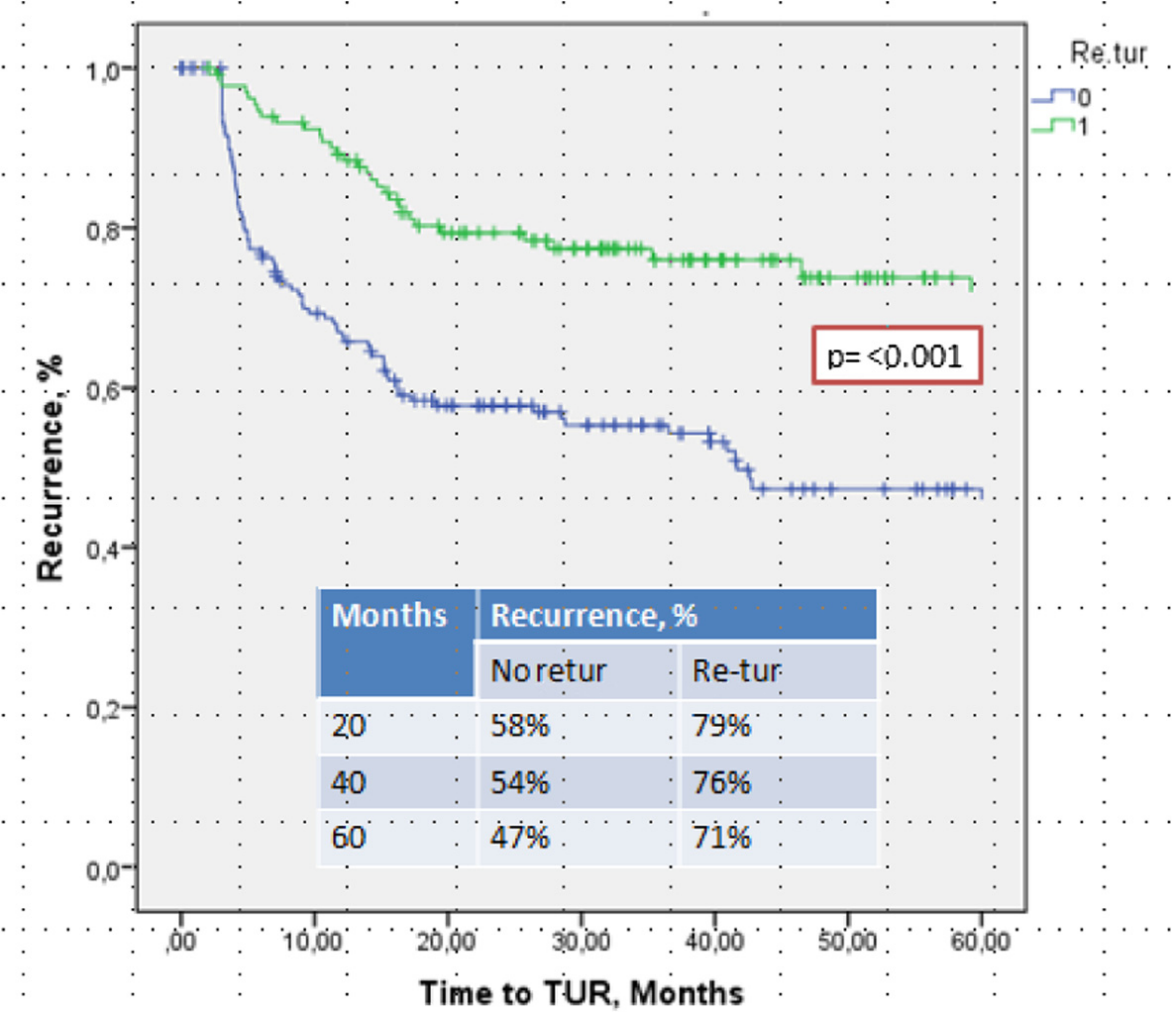
Recurrence-free survival was significantly better (*p* < 0.001) in the group that underwent reTUR

After a mean follow-up of 46 months, 115 patients (36.6 %) had experienced a recurrence, and 33 (10.5 %) a progression. The recurrence-free survival rate was significantly better (*p* < 0.001) in the group that underwent reTUR (Fig. 4).

**Fig. 5:**
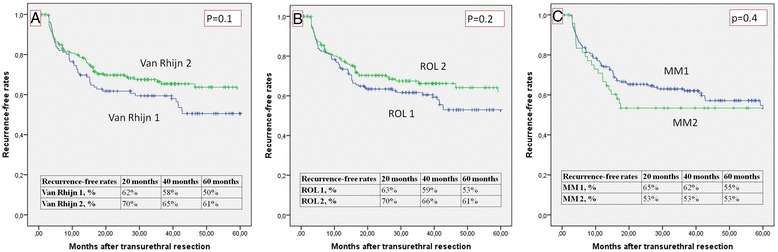
No significant correlations between the three substaging systems and the recurrence-free rate on Kaplan Meier estimates

**Fig. 6:**
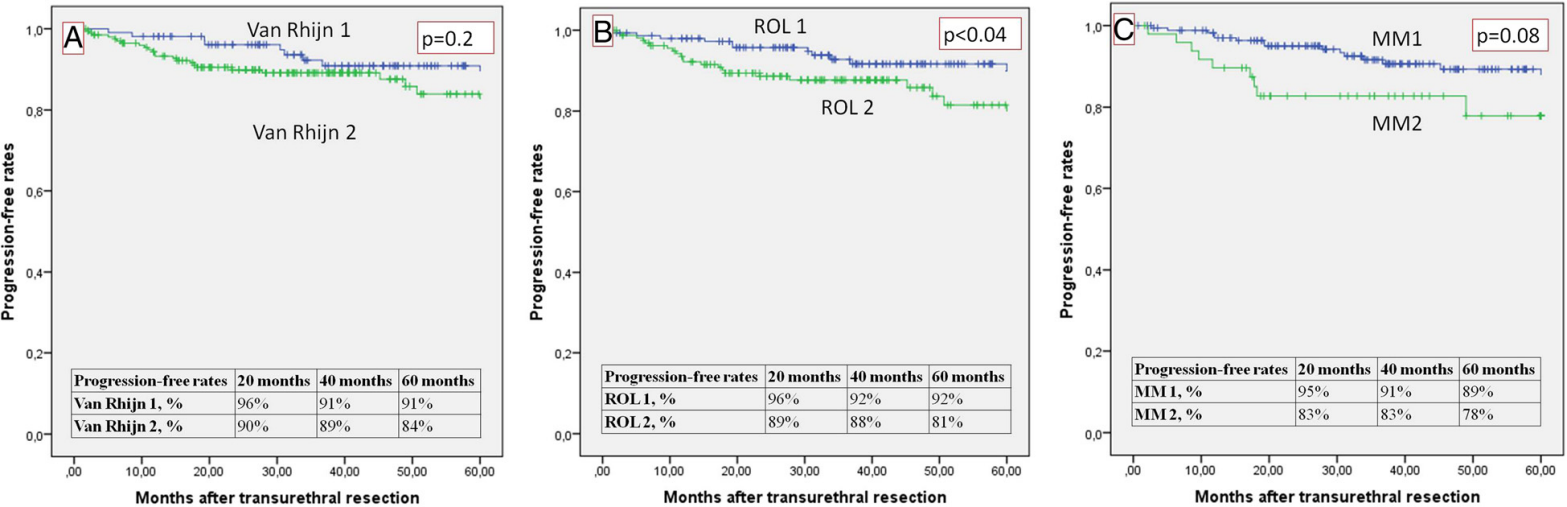
Progression-free rate: ROL substaging shows a significant correlation with progression in the Kaplan Meier estimates

**Fig. 7:**
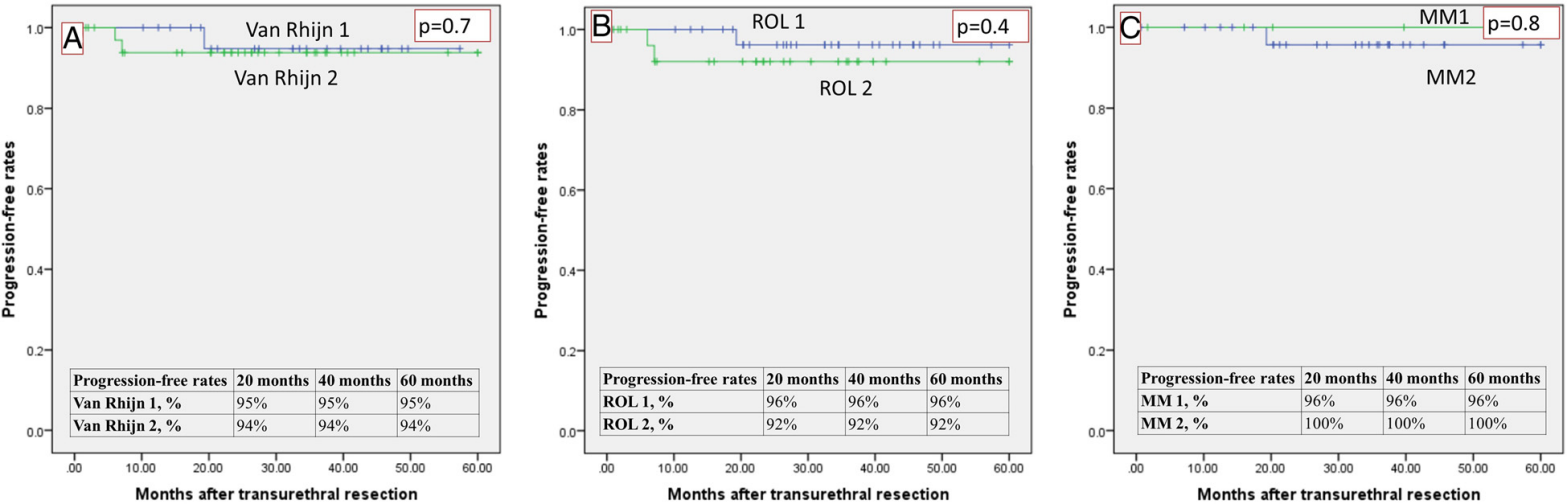
None of the three methods were able to predict progression in the group of 64 cases not treated with reTUR

**Fig. 8:**
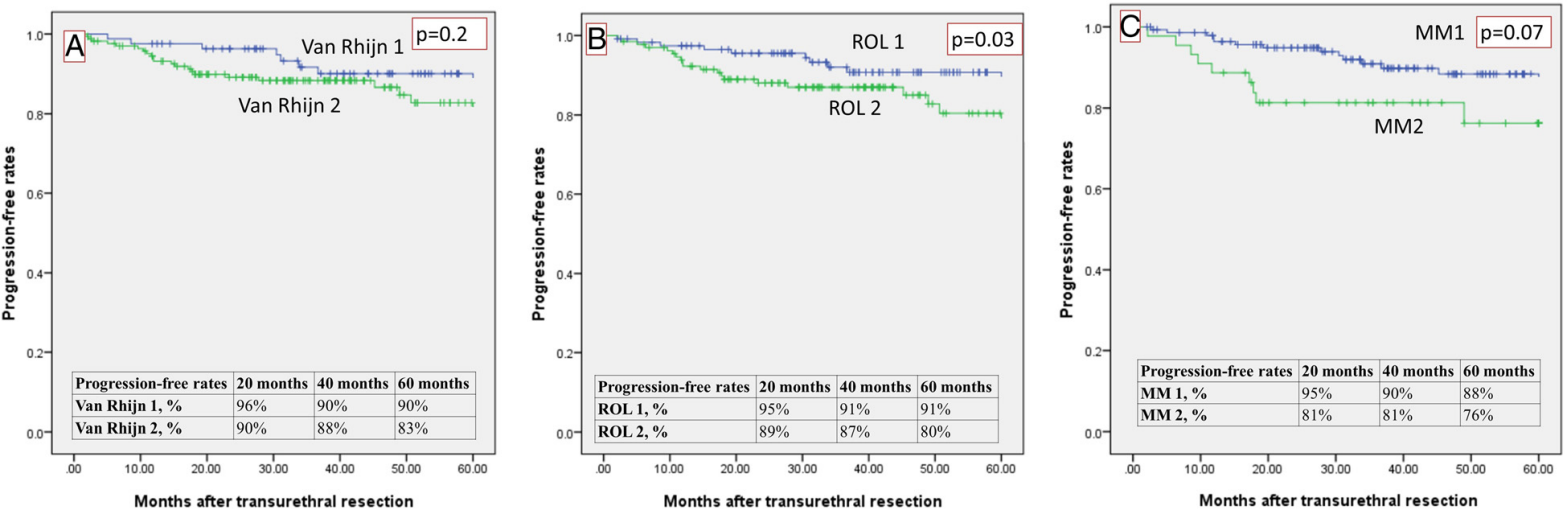
Progression-free rate: ROL substaging shows a significant correlation with progression in the Kaplan Meier estimates for the 250 cases treated with reTUR

The overall recurrence-free rates (RFR) are shown in Fig. 5. They were: 53 and 61 % for ROL1 and ROL2, respectively; 55 and 53 % for T1a and T1b, respectively; and 50 and 61 % for van Rhijn T1m and T1e, respectively. The overall progression-free rates (PFR) are given in Fig. 6. They were: 92 and 81 % for ROL1 and ROL2, respectively (*p* < 0.04); 89 and 78 % for T1a and T1b, respectively; and 91 and 84 % for van Rhijn T1m and T1e, respectively. Figure 7 shows the PFR for the 64/314 patients who had no reTUR, and Fig. 8 shows the PFR for the 250/314 who underwent reTUR; a statistically significant difference (*p* < 0.03) emerged between ROL1 and ROL2.

The results of univariate Cox’s regression analysis (including CIS and vascular neoplastic invasion) are given in Table 2: the correlations between the mixed transitional histology and a lower recurrence rate, and between ROL substaging and progression reached statistical significance.

**Table 2:** Univariate Cox regression analyses assessing recurrence and progression after transuretral resection due to T1HG bladder cancer

Variables	Univariate Recurrence		Univariate Progression	
	HR (IQR)	*P* value	HR (IQR)	*P* value
Age, years	0.99 (0.98–1.01)	0.5	1.00 (0.97–1.04)	0.8
ROL 2 *vs* ROL 1	0.81 (0.56–1.18)	0.2	2.09 (1.01–4.32)	0.04
T1e *vs* T1m	0.77 (0.53–1.11)	0.1	1.68 (0.78–3.63)	0.2
T1b *vs* T1a	1.25 (0.78–2.01)	0.4	2.06 (0.91–4.67)	0.08
CIS	0.77 (0.66–1.75)	0.7	1.44 (0.62–3.37)	0.4
LVI	1.11 (0.62–1.98)	0.7	2.55 (1.10–5.89)	0.03
Pure Transtitional vs. non pure transitional	0.40 (0.16.0.97)	0.04	0.31 (0.04–2.29)	0.2

## Discussion

Accurately staging bladder carcinoma is extremely important for prognostic purposes and treatment decision-making. The conclusion reached by the largest meta-analysis ever conducted on prognostic factors in high-grade T1 bladder carcinoma, recently published by W. Martin-Doyle et al. in the *Journal of Clinical Oncology,* was that depth of invasion is the prognostic factor with the greatest impact (HR: 3.55) and should be considered for inclusion among the TNM staging criteria [18]. Awareness of its potential clinical relevance and the ongoing lively debate on this issue convinced us to undertake the present study on 314 cases of T1 high-grade urothelial bladder carcinoma.

The first point worth emphasizing is the greater feasibility of the ROL and van Rhijn substaging approaches vis-à-vis T1a/b substaging: virtually all cases could be substaged using the former. This raises the question of why these systems do not suffer from the shortcomings of the T1a/b approach. The answer is probably that both the fragmentation and the modest orientability of TURB material often precludes any perpendicular definition of invasive growth and consequent anatomy-based T1a/b substaging. In addition, the *muscularis mucosae* and the vascular plexus that might be considered in lieu of it [16], are sometimes both absent.

We adopted a HPF-based approach of 20x objective to measure depth of invasion, which identified 152 cases (47.8 %) of minimally/initially invasive tumors (ROL1), i.e. fewer cases than those identified with the deeper T1a anatomy-based threshold (177 cases) and more than the number obtained with the shallower threshold proposed by van Rhijn as T1m (109 cases, 34 %). Our choice of a threshold at 1 mm of invasion was based on three points: *a*) a more balanced numerical distribution between the two subgroups; *b*) concern that the depth adopted by van Rhijn was too shallow, given the uneven thickness of the lamina propria, which reaches up to 3 mm in the cupola [16]; and *c*) concern that the anatomy-based T1a/b substaging might be too deep (we were able to split T1a cases into two groups: ROL1/T1a: 152 cases and ROL2/T1a: 25 cases).

Histological predictors of progression in high-grade tumors would be highly desirable, and the thresholds adopted by van Rhijn et al. [13] in their study proved to be the only significant independent predictors of progression and disease-specific survival on multivariate analysis.

When compared with the other substaging systems, our ROL approach seemed a promising threshold for predicting progression, much more feasible than the T1a/b system, and the only cutoff proving statistically significant on Kaplan Meier estimates (*p* < 0.04) (Fig. 6). The correlation between ROL substaging and progression reached statistical significance on univariate Cox’s regression analysis (Table 2).

Not surprisingly, none of the three substaging systems reliably predicted recurrence (p > 0.05), probably because of the important role of reTUR (Fig. 5). Although multifocality is a common finding in bladder cancer, and both low-grade and high-grade carcinomas may recur, these recurrences are usually local - as in our sample, where the recurrence-free survival rate was significantly better (*p* < 0.001) in the group that underwent reTUR (Fig. 4). The contribution of reTUR to final substaging is significant in terms of PFR too. None of the three methods were able to predict progression in the group of 64 who had no reTUR (Fig. 7), whereas the ROL again reached statistical significance in the Kaplan Meier estimates (*p* < 0.03) in the group of 250 patients who underwent reTUR (Fig. 8).

Several studies have addressed the issue of bladder cancer substaging in the past [10, 11, 19]. The study published by Cheng [19], one of the most often quoted in the subsequent literature, defines a depth of invasion of 1.5 mm as clinically relevant. Given the previously-mentioned uneven thickness of the lamina propria [16], this threshold is probably too deep, particularly in the trigone. Some whole-tumor chips of TUR material may contain detrusor muscle fibers that are not easy to detect without special immunostains like smoothelin [20]. Many studies might consequently include a variable proportion of understaged pT2 cases [18].

The anatomical approaches adopted by other authors [10, 11] suffer from the above-mentioned shortcomings, so the studies conducted by Bertz [14], Chang [12] and van Rhijn [13] represent a significant step forward, having ratified a quantitative microscopic assessment and also a shallower depth of invasion than the 1.5 mm threshold.

In the Chang et al. study [12], for instance, patients with high-grade carcinoma invading > 1 mm had a significantly worse outcome in terms of progression. These authors adopted the criterion of combining the sizes of individual foci of invasion, as in the present study, but they did not consider any early reTUR, which guarantees a complete substaging. In the study by van Rhijn et al. [13], on the other hand, the number of patients undergoing early reTUR is not stated and any cases of residual T1 were apparently not submitted to the same substaging procedure.

Concerning the limited prognostic relevance of any carcinoma in situ (CIS) in our study, it should be stressed that the diagnosis of CIS could be based on its detection in the peritumoral area, at the base of the papillae, or in any suspicious flat areas identified on cold biopsy. Such different approaches might lead to true CIS going underdetected, “*diluting any observed effect of reported CIS toward the null*” [18]. A better standardization of its definition could help unravel the question of its relevance. The weak prognostic value of lymphovascular invasion (LVI) in our study could be influenced by its relatively low incidence (10 % in our sample). Also in the study of F. Brimo et al. [21] about the value of millimetric invasion in T1 bladder carcinoma, LVI and concomitant CIS were less important than T1 depth of invasion (recorded by the Authors only in properly oriented specimens).

Our study has some limitations. First of all, patients’ data were retrieved retrospectively from our database, so our results may potentially be affected by all the limitations deriving from a retrospective design, including the fact that not all patients (79 %, 250/314) underwent reTUR. Second, no multivariate models were included in our analysis: this was a consequence of our limited cohort. Third, few progressions were recorded and this reduces the power of the study. Our progression rate was lower than in historical series published in the literature and this might be explained by the grade migration induced by the WHO 2004 Classification, and by the fact that 79 % of our patients underwent reTUR. Finally, the time frame between the first and second TURB procedures was as long as 3 months, and the total number of BCG instillations per patient was not taken into account.

As a final comment, it has been claimed that *en bloc* resection can overcome the drawbacks of conventional TURB [22]. Although tumor size and site might sometimes make *en bloc* resection difficult, removing the whole lesion would offer the following advantages: easier T1 substaging thanks to the specimen’s orientability; definition of the margins of resection (marked with ink), providing an indication of microscopic distance; significantly reduced seeding phenomena and a consequent reduction of early recurrences [23] and reTUR procedures. After all, from a surgical pathologist’s point of view, it is a matter of affording papillary urothelial carcinoma of the bladder the same dignity as other small surgical specimens, such as intestinal polyps or cervical conizations.

## Conclusion

In conclusion, although the international uropathology community [9] suggests that pT1 substaging would provide information important to clinicians, the histological approach has yet to be standardized. We believe, however, that a pure quantitative assessment of the extent of tumor invasion is promising and “user-friendly” and that 1 mm of invasion might represent a clinically useful threshold.

## References

[1] John E, Guido S, Jonathan E, Sesterhenn IA, WHO Classification of Tumours (2004). Pathology and Genetics of Tumours of the Urinary System and Male Genital Organs.

[2] Kulkarni GS, Hakenberg OW, Gschwend JE, Thalmann G, Kassouf W, Kamat A (2010). Zlotta. An updated critical analysis of the treatment strategy for newly diagnosed high-grade T1 (previously T1G3) bladder cancer. Eur Urol.

[3] Malavaud B (2004). T1G3 bladder tumours: the case for radical cystectomy. Eur Urol.

[4] Nigwekar P, Amin MB (2008). The many faces of urothelial carcinoma: an update with an emphasis on recently described variants. Adv Anat Pathol.

[5] Jimenez RE, Gheiler E, Oskanian P, Tiguert R, Sakl W, David P (2000). Grading the invasive component of urothelial carcinoma of the bladder and its relationship with progression-free survival. Am J Surg Pathol.

[6] Cho KS, Seo HK, Joung JY, Park WS, Ro JY, Han KS (2009). Lymphovascular invasion in transurethral resection specimens as predictor of progression and metastasis in patients with newly diagnosed T1 bladder urothelial cancer. J Urol.

[7] Palou J, Rodríguez-Rubio F, Millán F, Algaba F, Rodríguez-Faba O, Huguet J (2009). Recurrence at three months and high-grade recurrence as prognostic factor of progression in multivariate analysis of T1G2 bladder tumors. Urology.

[8] AJCC Cancer Staging Manual VII ed. Springer 2010.

[9] Amin MB, McKenney JK, Paner GP, Hansel DE, Grignon DJ, Montironi R (2013). International consultation on urologic disease - European Association of Urology Consultation on Bladder Cancer 2012. Eur Urol.

[10] Orsola A, Trias I, Raventós CX, Español I, Cecchini L, Búcar S (2005). Initial high-grade T1 urothelial cell carcinoma: feasibility and prognostic significance of lamina propria invasion microstaging (T1a/b/c) in BCG-treated and BCG-non-treated patients. Eur Urol.

[11] Angulo JC, Lopez JI (1997). The importance of the depth of invasion in stage T1 bladder carcinoma: a prospective cohort study. J Urol.

[12] Chang WC, Chang YH, Pan CC (2012). Prognostic significance in substaging of T1 urinary bladder urothelial carcinoma on transurethral resection. Am J Surg Pathol.

[13] van Rhijn BW, van der Kwast TH, Alkhateeb SS, Fleshner NE, van Leenders GJ, Bostrom PJ (2012). A new and highly prognostic system to discern T1 bladder cancer substage. Eur Urol.

[14] Bertz S, Denzinger S, Otto W, Wieland WF, Stoehr R, Hofstaedter F (2011). Substaging by estimating the size of invasive tumour can improve risk stratification in pT1 urothelial bladder cancer - evaluation of a large hospital-based single-centre series. Histopathology.

[15] Cheng L, Lopez-Beltran A, Bostwick DG. Bladder Pathology, Chapter 120. New Jersey, US: Wiley -Blackwel. 2012;194–213.

[16] Paner GP, Ro JY, Wojcik EM, Venkataraman G, Datta MW, Amin MB (2007). Further characterization of the muscle layers and lamina propria of the urinary bladder by systematic histologic mapping: implications for pathologic staging of invasive urothelial carcinoma. Am J Surg Pathol.

[17] Compérat E, Egevad L, Lopez-Beltran A, Camparo P, Algaba F, Amin M (2013). An interobserver reproducibility study on invasiveness of bladder cancer using virtual microscopy and heatmaps. Histopathology.

[18] Martin-Doyle W, Leow JJ, Orsola A, Chang SL, Bellmunt J (2015). Improving selection criteria for early cystectomy in high-grade T1 bladder cancer: a meta-analysis of 15,215 patients. J Clin Oncol.

[19] Cheng L, Neumann RM, Weaver AL, Spotts BE, Bostwick DG (1999). Predicting cancer progression in patients with stage T1 bladder carcinoma. J Clin Oncol.

[20] Paner GP, Brown JG, Lapetino S, Nese N, Gupta R, Shen SS (2010). Diagnostic use of antibody to smoothelin in the recognition of muscularis propria in transurethral resection of urinary bladder tumor (TURBT) specimens. Am J Surg Pathol.

[21] Brimo F, Chenbo W, Zeizafoun N, Tanguay S, Aprikina A, Mansure JJ (2013). Prognostic factors in T1 bladder urothelial carcinoma: the value of recording millimetric depth of invasion, diameter of invasive carcinoma, and muscolaris mucosae invasion. Hum Pathol.

[22] Ukai R, Hashimoto K, Iwasa T, Nakayama H (2010). Transurethral resection in one piece (TURBO) is an accurate tool for pathological staging of bladder tumor. Int J Urol.

[23] Kramer MW, Rassweiler JJ, Klein J, Martov A, Baykov N, Lusuardi L, et al. En bloc resection of urothelium carcinoma of the bladder (EBRUC): a European multicenter study to compare safety, efficacy, and outcome of laser and electrical en bloc transurethral resection of bladder tumor. World J Urol. 2015 Apr 25. [Epub ahead of print]. 10.1007/s00345-015-1568-6 25910478

